# Comparative efficacy of different dietary interventions for cardiopulmonary fitness at high altitude: a systematic review and network meta-analysis

**DOI:** 10.3389/fnut.2025.1658950

**Published:** 2025-11-04

**Authors:** Bin Wang, Yanle Xiong, Ning Lin, Jiaojiao Shi, Bo Zou, Xin Ma, Kaihong Zeng, Chao Kang

**Affiliations:** ^1^Department of Clinical Nutrition, Longgang Centre Hospital of Shenzhen, Shenzhen, Guangdong, China; ^2^Li Ka Shing Faculty of Medicine, The University of Hong Kong, Hong Kong, Hong Kong SAR, China; ^3^Department of Clinical Nutrition, The General Hospital of Western Theater Command, Chengdu, Sichuan, China; ^4^Department of Rehabilitation Medicine, The General Hospital of Western Theater Command, Chengdu, Sichuan, China; ^5^Department of Health Management Center & Institute of Health Management, Sichuan Provincial People's Hospital, University of Electronic Science and Technology of China, Chengdu, China

**Keywords:** high-altitude, nutritional interventions, cardiopulmonary fitness, network meta-analysis, randomized controlled trial

## Abstract

**Background:**

A plateau hypoxic environment can increase the physiological burden on athletes. Although nutritional interventions have been recognized as a potential strategy to improve plateau acclimatization, evidence in support of specific dietary patterns is still lacking. This study compared the effects of different dietary interventions on cardiopulmonary fitness during plateau exercise through systematic evaluation and network meta-analysis methods.

**Methods:**

This study systematically reviewed relevant literature up to June 2025 and included 20 randomized controlled trials (RCTs) conducted at altitudes above 1,500 meters involving healthy participants aged 16 years and above who engaged in physical activities. The primary outcomes included cardiopulmonary indicators [maximal oxygen uptake (VO_2max_), heart rate (HR)], blood biomarkers [peripheral oxygen saturation (SpO_2_), hematocrit (HCT)], and subjective perception indicators [rating of perceived exertion (RPE)]. For each outcome, the pooled effects of each intervention compared to others were estimated. Mean difference (MD) or standardized mean difference (SMD) with 95% Credible Intervals (95% CrI) were calculated. The Surface Under the Cumulative Ranking Curve (SUCRA) was used to rank the dietary interventions and quantify their relative effectiveness. In addition, the Grading of Recommendations Assessment, Development and Evaluation (GRADE) approach was applied to assess the quality of evidence.

**Results:**

A total of 20 randomized controlled trials involving 329 participants were included, evaluating eight dietary interventions. Moderate-quality evidence indicated that carbohydrate supplementation significantly improved the percentage of maximal oxygen uptake (VO_2max_) compared to placebo (SMD = 1.13, 95% CrI: 0.25 to 2.05) and reduced RPE scores (MD = −0.77, 95% CrI: −1.83 to −0.09). Moderate-quality evidence indicated that carbohydrate supplementation combined with glutamine ranked highest in improving SpO_2_ (SUCRA 84.54%) and RPE (SUCRA 69.37%), while iron supplementation showed the highest SUCRA rankings for HR (56.54%) and HCT (66.67%). However, these interventions did not demonstrate statistically significant advantages. Notably, the observed increase in VO_2max_ exceeded the minimally clinically important difference (MCID) of 1.0 ml/kg/min reported in previous studies, suggesting that the effect of carbohydrate supplementation on VO_2max_ may have clinical relevance.

**Conclusions:**

Differences exist in the effects of different dietary interventions on cardiopulmonary fitness during altitude exercise. The current network meta-analysis indicates that carbohydrate-based strategies show beneficial effects, with carbohydrate plus glutamine supplementation demonstrating greater advantages in SpO_2_ and RPE, while carbohydrate alone is more supported in improving VO_2max._ Therefore, carbohydrate-based strategies may serve as effective options to promote altitude acclimatization, whereas iron supplementation may have potential benefits in improving HCT and HR.

**Systematic review registration:**

https://www.crd.york.ac.uk/prospero/display_record.php?ID=CRD420251069629, identifier: CRD420251069629.

## 1 Introduction

In recent years, a growing number of people have been engaging in physical activity at high altitudes for purposes such as work, recreation, and sports (1). Particularly in the field of sports, interest in understanding the effects of altitude on physical performance has significantly increased since the 1968 Olympic Games were held at an altitude of over 2,000 meters (2). At present, high-altitude training has become a crucial method for enhancing endurance in athletic disciplines. The unique geographical and climatic conditions at high altitudes lead to a series of physiological adaptations in the human body. As altitude increases, the oxygen content in the air decreases, creating a hypoxic environment that induces physiological responses to enhance oxygen transport and utilization. Specifically, the body stimulates the expression of erythropoietin (EPO) through hypoxia-inducible factors (HIFs) in the kidneys. Once released into the bloodstream, EPO promotes the differentiation of erythroid progenitor cells in the bone marrow into mature red blood cells. In addition, hypoxia can increase capillary density and enhance mitochondrial efficiency. Together, these adaptations improve the oxygen-carrying capacity of the blood (3, 4). Studies have shown that once athletes adapt to high-altitude conditions, hypoxia regulation may benefit and protect the cardiovascular system, increase maximal oxygen uptake (VO_2max_), and thereby improve athletic performance (5).

Nutritional supplementation is crucial for high-altitude training. The International Olympic Committee (IOC) Nutrition Expert Group recommends that athletes increase their intake of energy, carbohydrate (CHO), iron, fluids, and antioxidant-rich foods during high-altitude training (6). Acute exposure to high altitudes for training imposes significant stress on various physiological and metabolic processes. A review on high-altitude nutrition, hydration and supplementation pointed out that prolonged stay at high altitude often leads to an imbalance between energy expenditure and intake, highlighting the importance of both macronutrient and micronutrient supplementation (7). This perspective is supported by several empirical studies. For example, a study on elite runners training at a 2,100-meter altitude camp found that daily supplementation with 200 mg of elemental iron significantly increased red blood cell mass following high-altitude exposure (8). Similarly, research by Oliver et al. showed that participants in high-altitude expeditions who received CHO supplementation experienced significantly lower Rated Perceived Exertion (RPE) and improved physical performance compared to the placebo group (9).

At present, there is no definitive evidence identifying which dietary intervention is most effective in improving cardiopulmonary fitness and physical performance during high-altitude exercise. Existing studies have primarily focused on the following nutritional interventions: nitrates, CHO, CHO combined with glutamine, high-protein diets, d-aspartic acid, iron, antioxidant-rich foods, rhodiola crenulata-and cordyceps sinensis (RC) (10–17). The aim of this study is to rank the effectiveness of different dietary and therapeutic interventions used to enhance cardiopulmonary function under high-altitude conditions by analyzing changes in cardiopulmonary indicators [(VO_2max_), heart rate (HR)], blood biomarkers [peripheral oxygen saturation (SpO_2_), hematocrit (HCT)], and RPE. The findings are intended to provide evidence-based guidance for athletes and high-altitude activity enthusiasts to optimize their nutrition and training strategies in such environments.

## 2 Methods

### 2.1 Search strategy

This systematic review and network meta-analysis followed the guidelines outlined in the Cochrane Handbook for Systematic Reviews of Interventions and the Preferred Reporting Items for Systematic Reviews and Meta-Analyses (PRISMA) statement (18). We conducted a comprehensive search across multiple online databases, including PubMed (Medline), Embase, and Web of Science–Science Citation Index, to identify potential studies. Additionally, manual searches were performed on preprint platforms (medRxiv and Research Square) as well as other databases such as CINAHL, China National Knowledge Infrastructure (CNKI), Wanfang Data, and Scielo. Although Scopus and ScienceDirect are also widely used in systematic reviews, they were not included in the present search. Scopus has substantial overlap with PubMed, Embase, and Web of Science, which were already comprehensively searched, while ScienceDirect primarily serves as a publisher-specific database and provides limited additional coverage beyond these sources. Therefore, the exclusion of these databases is unlikely to have materially affected the comprehensiveness of our literature search. Besides, to supplement the academic database searches, we also explored gray literature sources including ProQuest Dissertations and Theses, EThOS, and ClinicalTrials.gov. The search terms used to identify eligible studies included combinations of keywords such as (“dietary intervention” OR “nutritional supplementation” OR “nutrient supplementation” OR “dietary strategy” OR “macronutrient” OR “micronutrient”), (“high altitude” OR “plateau” OR “highland” OR “mountain” OR “hypoxia” OR “low oxygen”), and (“exercise performance” OR “physical performance” OR “physical training” OR “endurance training” OR “strength training”). Validated filters were applied to identify randomized controlled trials (RCTs). The search covered literature published from the inception of each database up to June 2025. The study protocol was registered in PROSPERO (CRD420251069629) and strictly followed throughout the review process.

### 2.2 Eligibility criteria

This study included randomized controlled trials (RCTs) involving healthy participants aged 16 years or older who engaged in physical activities (such as hiking, long-distance running, mountaineering, or cycling) at high-altitude regions above 1,500 meters (19). Healthy participants were defined as individuals without a history of acute mountain sickness, without cardiovascular, metabolic, pulmonary diseases, or other chronic conditions, and not taking concomitant medications that could affect exercise performance or altitude adaptation (20). All included studies were required to implement clearly defined dietary interventions and report at least one of the following outcome indicators: VO_2max_, HR, SpO_2_, HCT, or RPE. Exclusion criteria were as follows: (1) non-RCT study designs; (2) animal or *in vitro* experiments; (3) participants with severe chronic diseases (e.g., cardiovascular disease or chronic mountain sickness) or acute infections; (4) interventions not involving nutritional components (e.g., pure pharmacological or oxygen therapy); (5) studies with missing key data or inaccessible full texts; and (6) duplicate publications.

### 2.3 Screening and data extraction

The literature screening and data extraction were conducted independently by two reviewers (Y.X., D.M.). After removing duplicates, the titles, abstracts, and full texts were reviewed in strict accordance with the inclusion and exclusion criteria. A standardized data collection form was designed and developed using Microsoft Excel. The extracted information included: basic publication details (title, authors, year, and country); study design; participant characteristics (sample size, age); intervention details (altitude exposure model, altitude level, type of physical activity, type and dosage of dietary intervention, and duration); outcome indicators (VO_2max_, HR, SpO_2_, HCT, and RPE). For studies with missing data, we contacted the corresponding authors via email to request the original data. If no response was received, the study was excluded.

### 2.4 Quality assessment

This study employed the Cochrane Risk of Bias 2.0 tool (ROB 2.0) to rigorously assess the methodological quality of the included RCTs (21). Two independent reviewers (Y.X. and B.W.) conducted a cross-assessment of each study across the following seven key domains: (1) random sequence generation; (2) allocation concealment; (3) blinding of participants and personnel; (4) blinding of outcome assessors; (5) completeness of outcome data; (6) selective outcome reporting; (7) other potential sources of bias. Each trial was ultimately classified as having a “low risk of bias,” “unclear risk of bias,” or “high risk of bias.” All risk of bias assessment results were documented in the study characteristics table using Review Manager software (version 5.4).

### 2.5 Synthesis methods

Network meta-analyses based on both Bayesian and frequentist approaches were conducted using R version 4.4.2. A graphical network plot was used to illustrate the comparative relationships among different dietary interventions, where each node represented a dietary strategy with its size proportional to the number of included studies, and the thickness of edges reflected the number of trials with direct comparisons. For continuous outcomes, standardized mean differences (SMDs) were calculated when measurement units were inconsistent across studies, and mean differences (MDs) were used when units were consistent. Effect estimates were reported with 95% credible intervals (CrIs) under the Bayesian framework. Random-effects models were applied, and model fitting was optimized via four-chain Monte Carlo simulations to generate posterior samples. At least 20,000 adaptive iterations were set to ensure model convergence, followed by 50,000 sampling iterations (22). Interventions were ranked using the Surface Under the Cumulative Ranking (SUCRA) curve, with values expressed as percentages ranging from 0 to 100%. Higher SUCRA values indicated better rankings of intervention effectiveness (23). SUCRA results were visualized using STATA version 17. Besides, in this study, between-study heterogeneity was assessed using the between-study variance (τ^2^, tau-squared). A τ^2^ value greater than 0.36 was considered indicative of substantial heterogeneity, in line with previously suggested thresholds (24). To test the robustness of the results, sensitivity analyses were performed by excluding studies with high risk of bias. The purpose of these analyses was to evaluate whether the exclusion of potentially influential studies significantly altered the overall estimates or rankings.

### 2.6 Publication bias and inconsistency analysis

Publication bias was assessed by comparing the funnel plots and performing Egger's test for each outcome. A symmetric distribution in the funnel plots combined with a non-significant Egger's test result (*P* > 0.05) indicated a low risk of publication bias. All analyses were conducted using the netmeta package in R software (version 4.4.2). Additionally, inconsistency was evaluated by comparing the Deviance Information Criterion (DIC) values between the consistency and inconsistency models (25). The model with the lower DIC was preferred. A DIC difference of ≥3 between the two models was considered to indicate a meaningful difference (26).

### 2.7 Confidence in estimates

We assessed the quality of evidence using the CINeMA (Confidence in Network Meta-Analysis) platform, which is based on the GRADE (Grading of Recommendations Assessment, Development and Evaluation) framework. CINeMA evaluates each comparison across six domains—within-study bias, reporting bias, indirectness, imprecision, heterogeneity, and incoherence—and integrates direct and indirect evidence to determine the overall confidence in network meta-analysis estimates. According to this approach, evidence from RCTs starts at “high” confidence but may be downgraded due to these limitations, resulting in ratings of “moderate,” “low,” or “very low.”

## 3 Results

### 3.1 Search and selection

**Figure 1:**
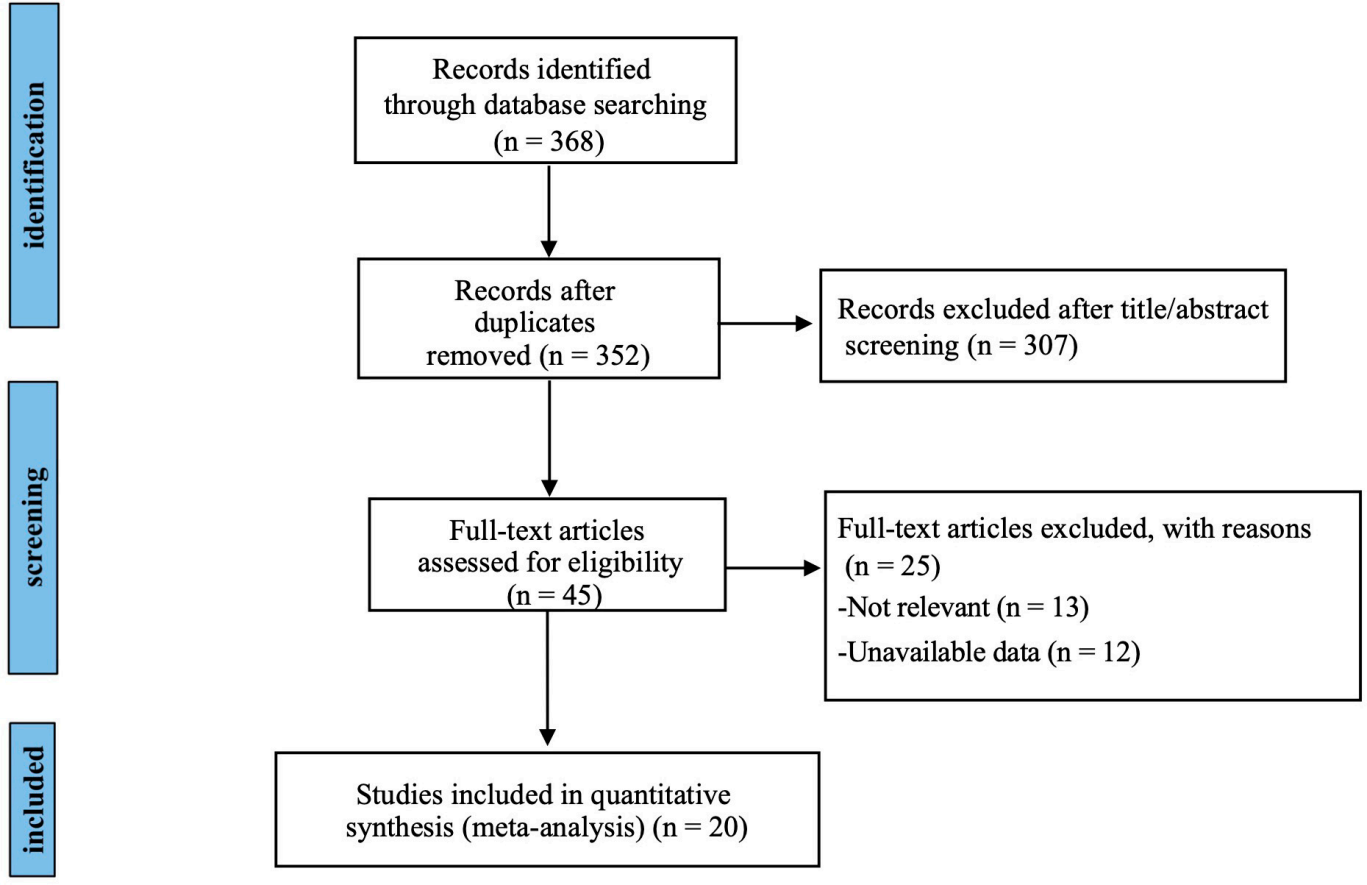
Flow diagram for identification of studies in the systematic review.

A total of 368 relevant studies were identified through systematic searching, and duplicate records were removed. The remaining 45 studies were assessed based on their titles and abstracts. After a full-text review, 13 studies were excluded because they did not align with the objectives of the meta-analysis, and 12 studies were excluded due to the absence of the outcome measures of interest. Ultimately, 20 RCTs with 329 participants were included (Figure 1).

### 3.2 Study characteristics

The baseline characteristics of the quantitative and qualitative studies are detailed in Table 1. This meta-analysis included 20 studies published between 1999 and 2023, involving eight types of dietary interventions. The proportion of participants in each dietary intervention was as follows: nitrate (48.94%), CHO (26.44%), antioxidant-rich foods (9.42%), CHO combined with glutamine (5.47%), RC (5.47%), d-aspartic acid (4.86%), iron (4.86%), and high-protein (2.74%). The study duration ranged from 10 days to 7 weeks.

**Table 1:** Characteristics of the studies included in this review.

**Author, Year**	**Country**	**Population**	**Sample size (*n*)**	**Age (years)**	**Altitude (m)**	**Exposure state**	**Sports mode**	**Research method**	**Intervention regimen**	**Intervention dose**	**Control regimen**	**Duration**	**Outcome indicator**
Friedmann, 1999 (15)	Germany	National German boxing team	16	C: 24.2 ± 2.9 P: 23.8 ± 2.6	1,800	Continuous altitude exposure	Boxing, training maximal strength, sprint training. endurance training, etc.	Randomized double-blind placebo-controlled design	Ferrous-glycine-sulfate (iron supplementation)	2 × 100 mg elemental iron daily (1,335 mg ferrous-glycine-sulfate)	Placebo (fructose in identical gelatin-coated capsules)	18 days	
Fulco, 2005 (30)	USA	Healthy males	16	p: 25.1 ± 6 C: 25.3 ± 6	4,300	Continuous altitude exposure	Cycling	Double-blind, placebo controlled prospective design	Carbohydrate [Tropical punch flavored blend of maltodextrin (mass·volume^−1^, 9%), glucose (2%), and fructose (1%)]	0.175 g·kg^−1^ body weight	Equal volume of indistinguishable PLA	10 days	
Oliver, 2012 (9)	UK	Healthy adults	17	C: 24 ± 4 p: 32 ± 11	5,192	Continuous altitude exposure	Mountaineering	A prospective, experimental, randomized parallel groups design	Carbohydrate (principally maltodextrin)	Provided 100 g·L^−1^ of carbohydrate and 370 kcal·L^−1^	Placebo solution was composed principally of natural flavorings and aspartame, and its nutrient content was negligible.	22 days	
Charlot, 2013 (13)	France	Healthy active male	11	18–25	3,500	Intermittent exposure at simulated altitude	Not mentioned	Single blind and in a counter balanced order	Carbohydrate (breakfasts consisted of high CHO)	2,340 kJ, 70% CHO, 12% protein	High-protein (35% CHO, 48% protein) breakfast	2 weeks	
Chen, 2014 (17)	China (Taiwan)	Long-distance track and field male athletes	18	19.66 ± 0.18	2,200	Continuous altitude exposure	Running, weight training, ball activity	Double-blind and placebo-controlled experimental de-sign	Rhodiola crenulata plus Cordyceps sinensis supplement (capsule)	1,000 mg: breakfast 1,000 mg: dinner	Starch placebo 1,000 mg: breakfast 1,000 mg: dinner	2 weeks	
Arnold, 2015 (10)	UK	Well-trained competitive male runners	10	37 ± 13	2,500	Intermittent exposure at simulated altitude	Running	Double-blind repeated measures crossover design	Concentrated beetroot juice	70 ml (7 mmol nitrates)	Same texture, taste and appearance with negligible nitrate concentration placebo	17 days	
Hennis, 2016 (27)	UK	Healthy male students	40	16 ± 1	5,300	Continuous altitude exposure	Climbing	Single-blind parallel group randomized controlled design	Concentrated beetroot juice	140 ml (10 mmol nitrates)	Concentrated blackcurrant juice	11 days	
Shannon, 2016 (60)	UK	Healthy males	12	24.4 ± 4.3	2,500	Intermittent exposure at simulated altitude	Running	Randomized double-blind placebo-controlled design	Concentrated beetroot juice	138 ml (15.2 mmol nitrates)	Placebo with negligible nitrates	6 weeks	
Rossetti, 2017 (61)	UK	Recreationally active males	20	22 ± 4	4,219	Intermittent exposure at simulated altitude	Weighted trekking	Randomized-double-blinded placebo-controlled crossover design.	Concentrated beetroot juice	70 ml (6.4 mmol nitrates)	Placebo with negligible nitrates	22 days	
Shannon, 2017 (32)	UK	Healthy males	10	23 ± 3	4,300	Intermittent exposure at simulated altitude	Running	Randomized controlled design	Concentrated beetroot juice	140 ml (12.5 mmol nitrates)	Placebo with negligible nitrate concentration	7 weeks	
Koivisto, 2018 (16)	Norway	Elite athletes	31	23 ± 5	2,320	Continuous altitude exposure	Different training plans swimming, triathlon and athletics, etc.	Randomized controlled trial	Antioxidant-rich foods	750 ml fruit-, vegetable- and berry smoothie, 50 g dried berries and fruits, 40 g walnuts, and 40 g dark chocolate (>70% cocoa content)	Eucaloric foods (4.2 MJ or 1,000 kcal) with a significantly lower antioxidant content	3 weeks	
Paris, 2019 (20)	USA	Endurance-trained men	12	25 ± 1	3,000	Intermittent exposure at simulated altitude	Running	Randomized double-blind placebo-controlled design	Carbohydrate	6% carbohydrate solution: 15 ml/kg/h	A non-caloric sweetener	4 weeks	
Kent, 2019 (29)	Australia	Moderately trained, male	12	22.3 ± 2.6	3,000	Intermittent exposure at simulated altitude	Cycling	Double blind, repeated-measures, counter-balanced design	Concentrated beetroot juice	2 × 70 ml (6.45 mmol nitrates)	Placebo with negligible nitrates	Not mentioned	
Robinson, 2020 (62)	UK	Trained males	8	23 ± 4	2,400	Intermittent exposure at simulated altitude	Running	Repeated-measures, crossover design	Concentrated beetroot juice	140 ml (12.4 mmol nitrates)	Placebo with negligible nitrates	4 weeks	
Caris, 2020 (12)	Brazil	Healthy and trained male	9	26.4 ± 3.5	4,300	Intermittent exposure at simulated altitude	Running	Randomized double-blind placebo-controlled design	(1) Carbohydrate (Maltodextrin) (2) Carbohydrate and glutamine	(1) 200 ml of 8% maltodextrin/ 20 min (2) 20 g/day for 6 days, 8% maltodextrin (200 ml/every 20 min)	Placebo (10 g starch + 10 g lactose)	24 days	
Bradbury, 2020 (11)	USA	Healthy males	14	Not mentioned	4,300	Continuous altitude exposure	Running	Randomized controlled design	Carbohydrate (65.25 g fructose + 79.75 g glucose)	Ingested at 1.8 g/min	PLA (*n* = 6, 4 standard and 2 high protein)	22 days	
Tavares-Silva, 2020 (31)	Brazil	Healthy and physically active male	8	24 ± 3	4,200	Intermittent exposure at simulated altitude	Running	Randomized double-blind placebo-controlled design	Carbohydrate (maltodextrina strawberry-flavored)	200 ml solution of carbohydrate	A placebo 0 kcal (strawberry-flavored Crystal Light)	2 weeks	
Marshall, 2021 (28)	UK	Healthy adults	22	28 ± 12	4,800	Continuous altitude exposure	Climbing	Single-blinded randomized control study	Concentrated beetroot juice	140 ml (12.5 mmol nitrates)	Non-nitrate, same calorie placebo	20 days	
Hennis, 2022 (1)	UK	healthy volunteers	27	21 males: 28.9 ± 5.2	4,559	Continuous altitude exposure	Cycling	Randomized, double blind, placebo-controlled factorial design	Concentrated beetroot juice	0.18 mmol/kg/day nitrates	Placebo with negligible nitrates	Not mentioned	
Płoszczyca, 2023 (14)	Poland	Male Boxers	16	18–25	2,500	Intermittent exposure at simulated altitude	Boxing drills, endurance, resistance exercises	Randomized Controlled Trial	D-aspartic acid	6 g/day, divided into two doses per day	Placebo (cellulose) in identical gelatin capsules	14 days	

Maximal oxygen uptake (VO_2max_); heart rate (HR); peripheral oxygen saturation (SpO_2_); rating of perceived exertion (RPE); hematocrit (HCT).

### 3.3 Subject characteristics

A total of 329 independent participants were included in this meta-analysis. Due to the use of a crossover design in some studies, all participants underwent both the intervention phase and the control phase, resulting in 441 participant data points, with 53.74% from the intervention group and 46.26% from the control group. The study population was predominantly male, with 80.17% in the intervention group and 75% in the control group. Regarding altitude distribution, 40.59% of the participant data points were from high-altitude areas above 3,500 meters. Among the different study locations, the highest proportion of participant data points came from the UK (49.20%). In terms of exercise modalities, running was the most common form of exercise, with 34.18% in the intervention group and 34.31% in the control group (Table 2).

**Table 2:** Demographic characteristics of participants.

**Characteristics**	**Intervention**	**Placebo**
Total numbers, *n* (% total)	237 (53.74%)	204 (46.26%)
**1. Gender**		
Males	190 (80.17%)	153 (75%)
Males + Females	47 (19.83%)	51 (25%)
**2. Altitude**		
< 3,500 m	91 (38.40%)	88 (43.14%)
>3,500 m	146 (61.60%)	116 (56.86%)
**3. Nation**		
UK	107 (45.15%)	110 (53.92%)
USA	28 (11.81%)	26 (12.75%)
Brazil	26 (10.97%)	17 (8.33%)
Norway	16 (6.75%)	15 (7.35%)
Australia	12 (5.06%)	12 (5.88%)
France	22 (9.28%)	0
China	9 (3.80%)	9 (4.41%)
Germany	9 (3.80%)	7 (3.43%)
Poland	8 (3.38%)	8 (3.92%)
**4. Sport modes**		
Running	81 (34.18%)	70 (34.31%)
Cycling	34 (14.35%)	34 (16.67%)
Climbing	32 (13.5%)	30 (14.71%)
Weighted trekking	20 (8.43%)	20 (9.80%)
Mountaineering	6 (2.53%)	11 (5.39%)
Mixed training	42 (17.72%)	39 (19.12%)
Not mentioned	22 (9.28%)	0
**4. Interventions**		
Nitrate	113 (47.68%)	111 (52.11%)
CHO	62 (26.16%)	54 (25.35%)
Antioxidant-rich foods	16 (6.75%)	15 (7.04%)
RC	9 (3.79%)	9 (4.23%)
CHO+Glutamine	9 (3.79%)	9 (4.23%)^*^
Fe	9 (3.79%)	7 (3.29%)
D-aspartic acid	8 (3.38%)	8 (3.76%)
High protein	11 (4.64%)	0

CHO, carbohydrate; Fe, iron; RC, rhodiola crenulata-and cordyceps sinensis.

^*^The 9 control group cases listed under CHO+Glutamine were part of studies that included three arms: CHO, CHO+Glutamine, and placebo. These control participants were used as a shared comparator for both intervention groups (CHO and CHO+Glutamine) and thus appear in both categories. This classification does not represent duplicate participants but reflects the structure of the original study design.

### 3.4 Risk of bias

**Figure 2:**
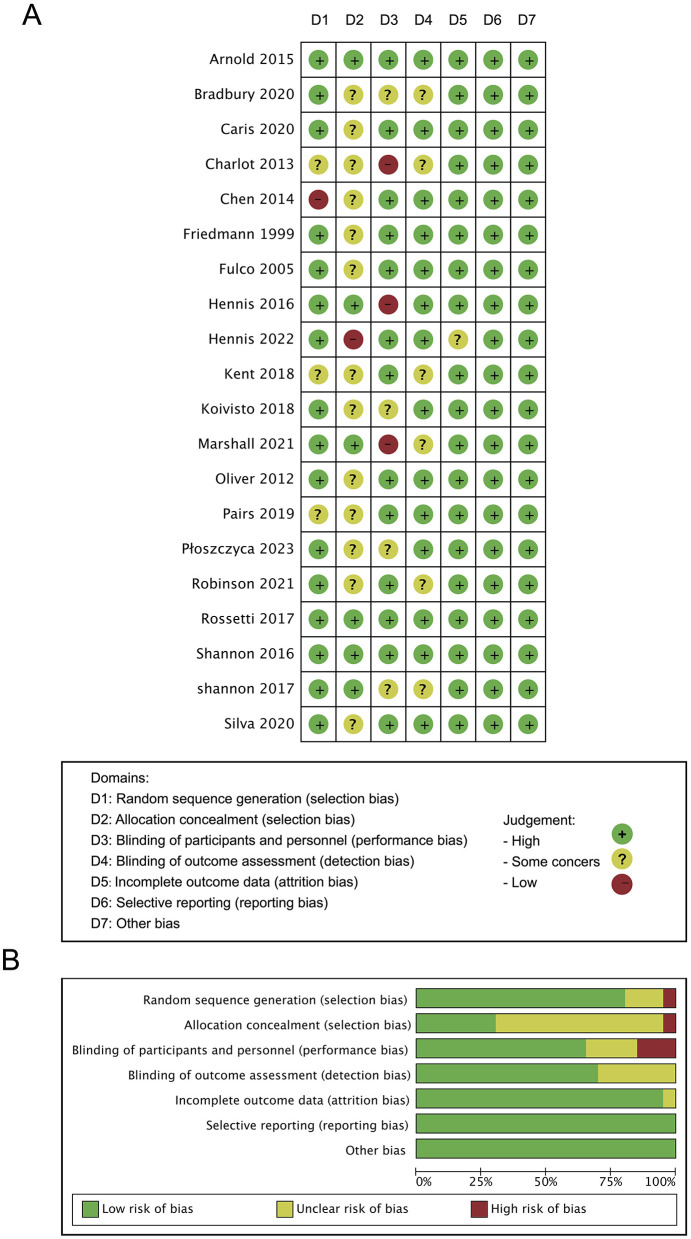
Risk of bias (ROB) analysis highlighting results in all domains examined within the nine identified studies **(A)** and overall risk of bias for included studies **(B)**.

Bias risk analysis of the 20 included studies was conducted using the RoB 2 tool (Figure 2). Five studies showed some high-risk points [Charlot et al. (13); Cheng, (17); Hennis et al. (27); Hennis et al. (1); Marshall et al. (28)]. Additionally, four studies had issues with random sequence generation [Charlot et al. (13); Cheng, (17); Kent et al. (29); Paris et al. (20)]. Fourteen studies had problems with allocation concealment [Bradbury et al. (11); Caris and Thomatieli-Santos (12); Charlot et al. (13); Cheng, (17); Friedmann et al. (15); Fulco et al. (30); Hennis et al. (1); Kent et al. (29); Koivisto et al. (16); Oliver et al. (9); Paris et al. (20); Płoszczyca et al. (14); Robison, (62); Tavares-Silva et al. (31)]. Seven studies had issues with blinding of participants and researchers [Bradbury et al. (11); Charlot et al. (13); Hennis et al. (27); Koivisto et al. (16); Marshall et al. (28); Płoszczyca et al. (14); Shannon et al. (32)]. Six studies had problems with blinding of outcome assessors [Bradbury et al. (11); Charlot et al. (13); Kent et al. (29); Marshall et al. (28); Robison, (62); Shannon et al. (32)].

### 3.5 Quantitative synthesis

**Figure 3:**
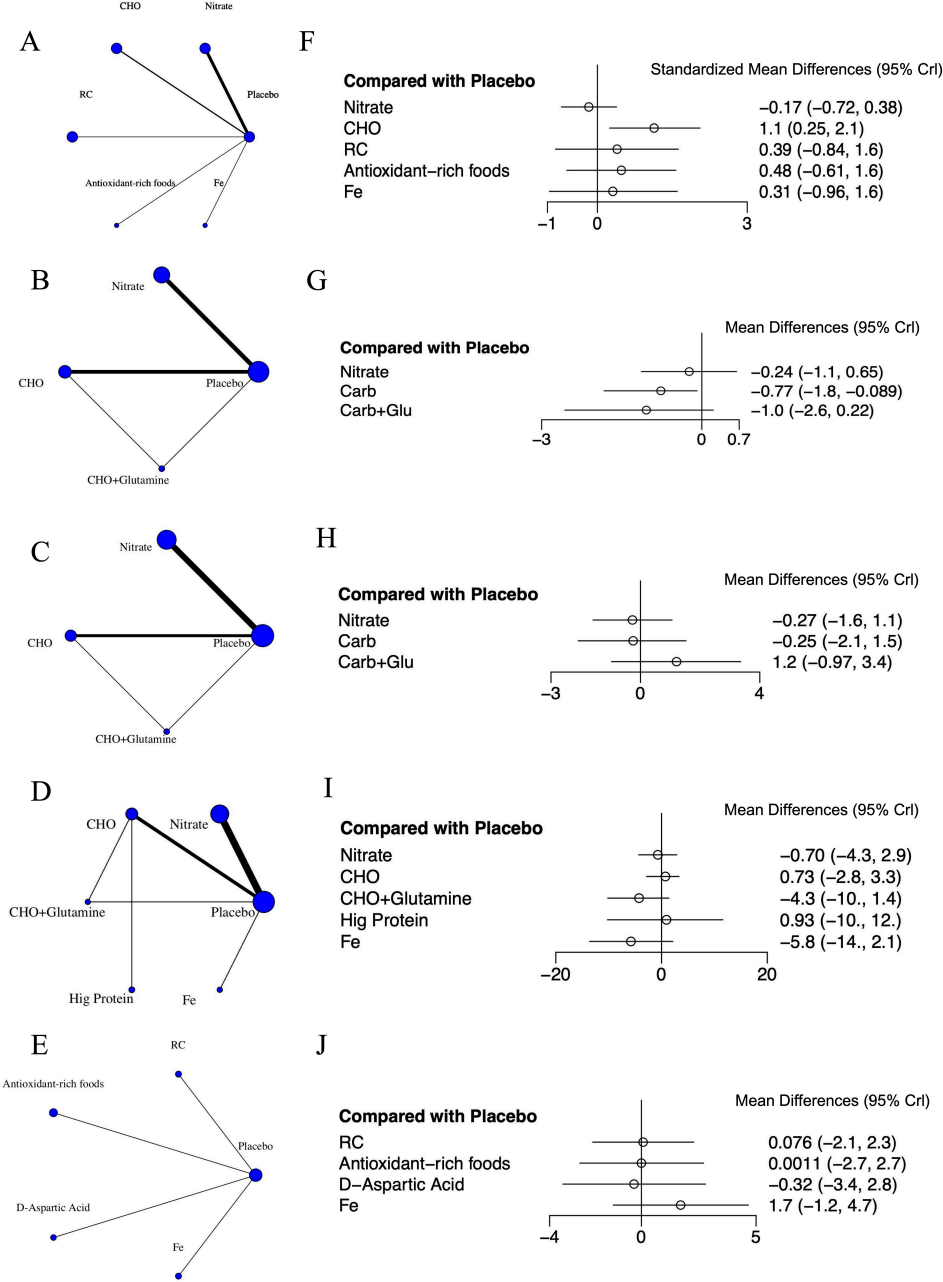
Network meta-analysis of various interventions on cardiopulmonary fitness. **(A)** Network of interventions for VO_2max_. **(B)** Network of interventions for RPE. **(C)** Network of interventions for SpO_2_. **(D)** Network of interventions for HR. **(E)** Network of interventions for HCT. **(F)** Forest plot displaying weighted standardized mean difference and 95% credible interval for the effect of various interventions vs. placebo on VO_2max_ levels. **(G)** Forest plot displaying weighted mean difference and 95% credible interval for the effect of various interventions vs. placebo on RPE levels. **(H)** Forest plot displaying weighted mean difference and 95% credible interval for the effect of various interventions vs. placebo on SpO_2_ levels. **(I)** Forest plot displaying weighted mean difference and 95% credible interval for the effect of various interventions vs. placebo on HR levels. **(J)** Forest plot displaying weighted mean difference and 95% credible interval for the effect of various interventions vs. placebo on HCT levels. CHO, carbohydrate; Fe, iron.

Figure 3 shows the network interventions for each outcome. The thickness of the lines corresponds to the number of studies, and the size of the nodes corresponds to the number of included treatment options. For RPE, SpO_2_, and HR, the networks contained at least one closed loop, enabling the assessment of inconsistency. In contrast, the VO_2max_ and HCT networks consisted only of star-shaped structures centered on the placebo, with no closed loops available for inconsistency checks.

### 3.6 Ranking of interventions

The SUCRA was used to calculate the probability of each dietary intervention being ranked for each outcome. The results are shown in Table 3.

**Table 3:** SUCRA ranking of dietary interventions for cardiopulmonary outcomes at high altitude.

	**SUCRA % (Rank)**											
**Dietary approaches**	**HR**			**SpO**_2_			**RPE**			**HCT**		
	**1**	**2**	**3**	**1**	**2**	**3**	**1**	**2**	**3**	**1**	**2**	**3**
Nitrate	2.20	13.20	32.63	6.29	22.50	28.56	4.82	14.94	54.19	–	–	–
CHO	0.23	2.03	9.02	4.09	28.70	25.04	25.69	61.68	11.64	–	–	–
RC	–	–	–	–	–	–	–	–	–	8.74	24.03	25.07
Antioxidant-rich foods	–	–	–	–	–	–	–	–	–	12.54	24.16	16.49
CHO+Glutamine	30.92	44.65	15.41	84.54	7.93	4.83	69.37	21.58	5.73	–	–	–
High protein	10.02	12.41	14.94	–	–	–	–	–	–	–	–	–
D-aspartic acid	–	–	–	–	–	–	–	–	–	10.48	18.73	13.08
Fe	56.54	25.32	8.98	–	–	–	–	–	–	66.67	16.82	7.05
Placebo	0.09	2.39	19.01	5.08	40.86	41.57	0.11	1.81	28.44	1.58	16.26	38.31

CHO, carbohydrate; Fe, iron; RC, rhodiola crenulata-and cordyceps sinensis.

### 3.7 Results of cardiopulmonary fitness

**Figure 4:**
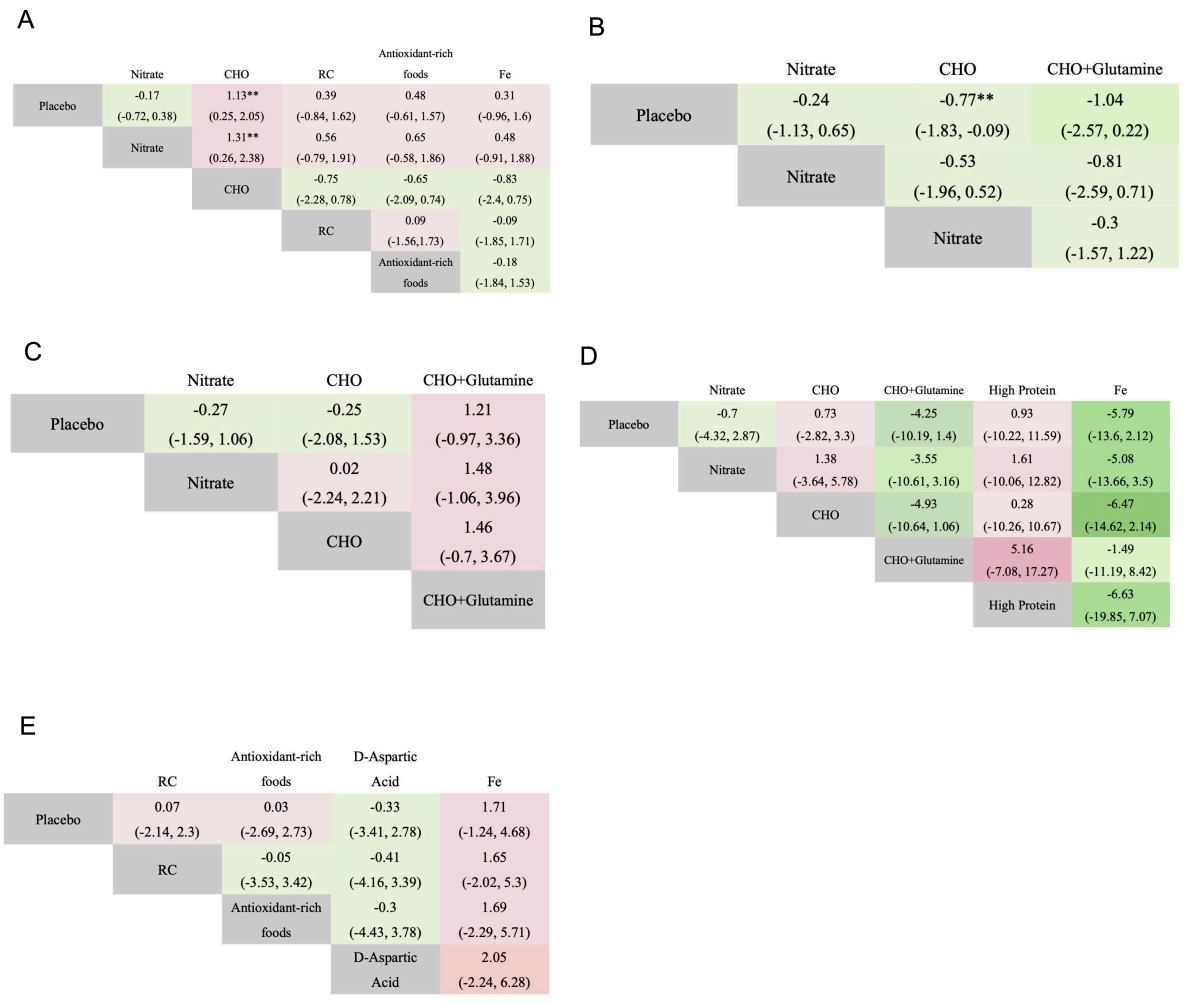
League table displaying pairwise comparisons among various interventions on VO_2max_ **(A)**, RPE **(B)**, SpO_2_ **(C)**, HR **(D)**, and HCT **(E)**. Statistically significant differences are bolded (**). CHO, carbohydrate; Fe, iron; **P < 0.01.

**Figure 5:**
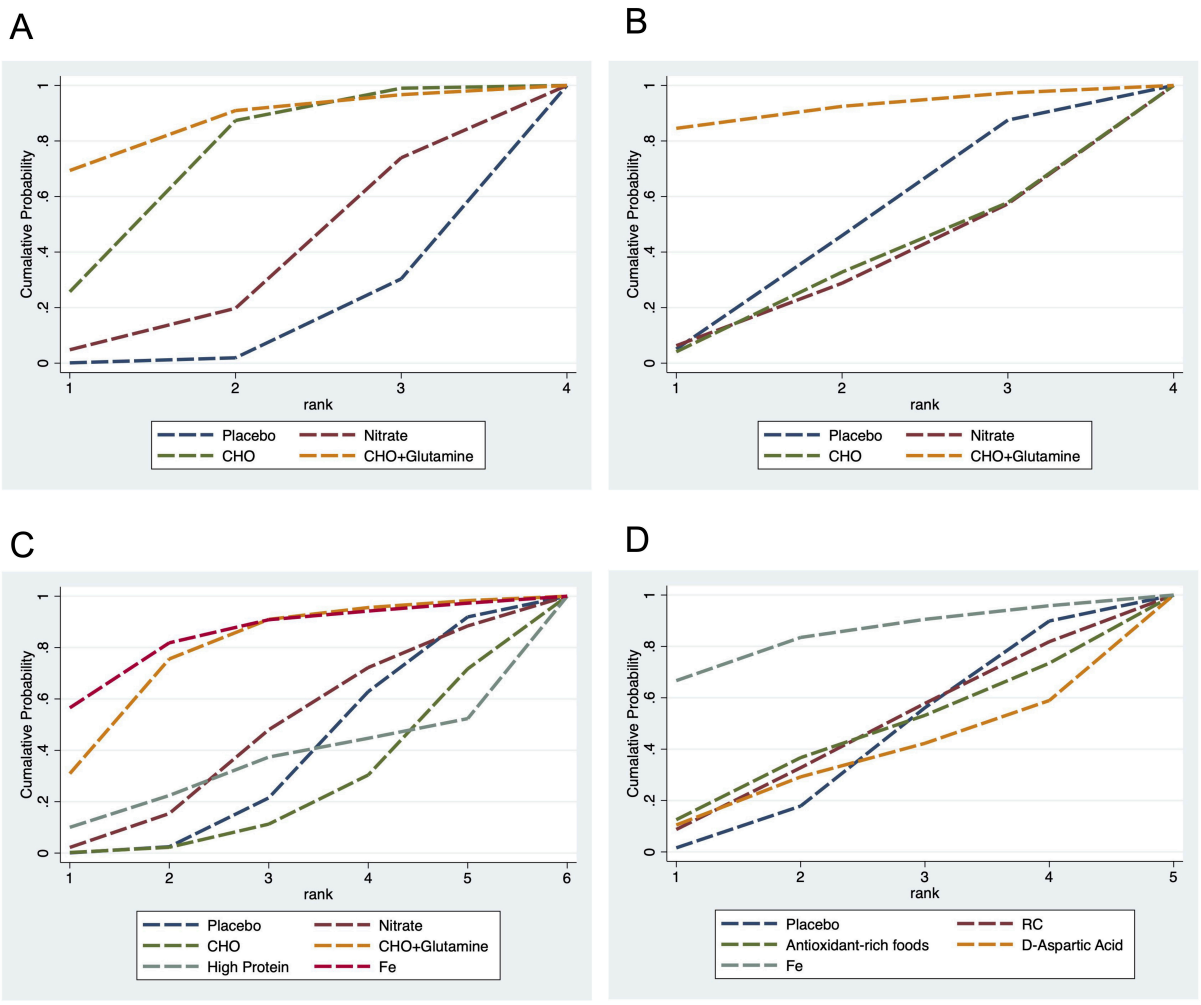
Surface Under the Cumulative Ranking Curve (SUCRA) illustrating the cumulative probability of each intervention being among the best in terms of RPE **(A)**, SpO_2_ **(B)**, HR **(C)**, and HCT **(D)** levels. CHO, carbohydrate; Fe, iron.

A total of five cardiopulmonary outcomes (VO_2max_, HR, SpO_2_, HCT, and RPE) were analyzed across multiple studies and interventions (Figure 3). CHO supplementation consistently showed favorable effects, demonstrating significant improvements in VO_2max_ compared to both placebo (SMD = 1.13, 95% CrI: 0.25–2.05) and nitrate supplementation (SMD = 1.31, 95% CrI: 0.26–2.38; Figure 4). For RPE, CHO alone also showed superiority over placebo (MD = −0.77, CrI: −1.83 to −0.09; Figure 4). For both RPE and SpO_2_, CHO combined with glutamine ranked highest in effectiveness (SUCRA: 69.37 and 84.54%, respectively; Figure 5). Besides, iron supplementation ranked highest for improving HR (SUCRA: 56.54%) and HCT (SUCRA: 66.67%; Figure 5).

### 3.8 Heterogeneity assessment

Between-study heterogeneity was evaluated using the estimated τ^2^ for each cardiopulmonary outcome. The τ^2^ values for VO_2max_ (τ^2^ ≈ 0.12) and RPE (τ^2^ ≈ 0.33) were below the commonly cited threshold of 0.36, indicating low heterogeneity (24). In contrast, the τ^2^ values for SpO_2_ (τ^2^ ≈ 0.59), HR (τ^2^ ≈ 2.03), and HCT (τ^2^ ≈ 0.71) exceeded this threshold, suggesting substantial heterogeneity in these outcomes. These results imply that the studies reporting VO_2max_ and RPE yielded more consistent findings, whereas greater variability was observed among studies evaluating SpO_2_, HR, and HCT.

### 3.9 Publication bias and hypothesis of overall consistency between networks

**Figure 6:**
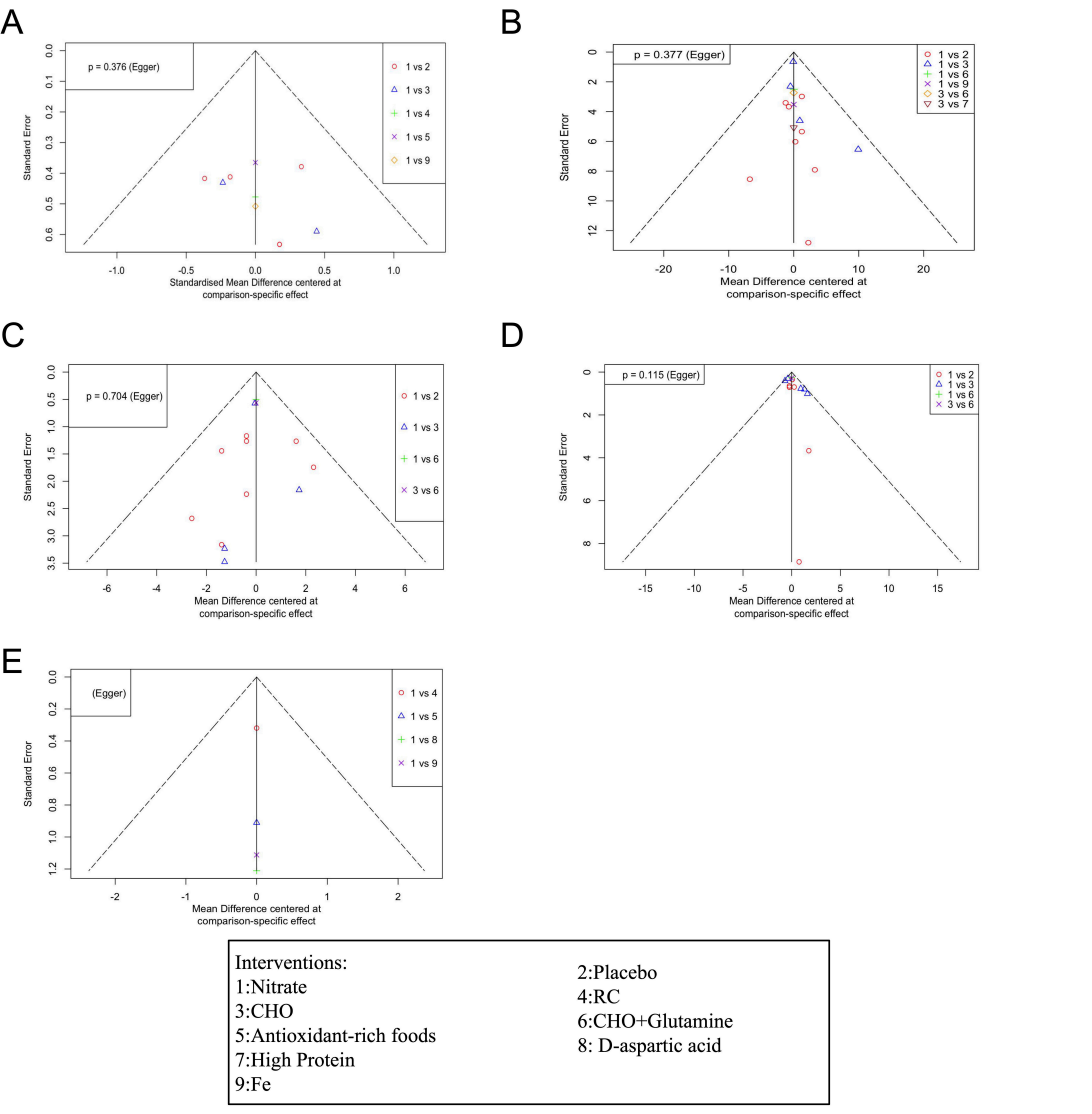
Funnel plot detailing publication bias in the studies reporting the impact of dietary nitrate on VO_2max_ **(A)**, HR **(B)**, SpO_2_ **(C)**, RPE **(D)**, and HCT **(E)** levels. CHO, carbohydrate; Fe, iron; RC, rhodiola crenulata- and cordyceps sinensi.

Figure 6 shows the funnel plot for publication bias. The results of the Egger regression test indicate no evidence of publication bias (all egger regression tests for comparisons were >0.05, Table 4). The comparison of DIC values between the consistency and inconsistency models showed that the consistency model was superior for all outcomes (Table 5), with no evidence of inconsistency in the network meta-analysis.

**Table 4:** Egger's test for publication bias in dietary interventions on cardiopulmonary outcomes at high altitude.

**Outcome**	***P-*value**
VO_2max_	0.3762
HR	0.3774
SpO_2_	0.7043
RPE	0.1147
HCT	–

**Table 5:** Deviance information criterion (DIC) comparison between consistency and inconsistency models for each outcome.

**Outcome**	**Inconsistency**	**Consistency**
VO_2max_	14.356	19.980
HR	57.962	43.721
S_P_O_2_	49.926	28.138
RPE	45.776	39.506
HCT	16.040	16.047

### 3.10 Confidence in evidence and sensitivity analyses

We used the CINeMA platform to systematically evaluate the quality of evidence for the outcomes of our network meta-analysis. Overall, most comparisons were rated as low to moderate confidence, mainly due to concerns about within-study bias and imprecision. Several indirect comparisons were further downgraded to very low confidence because of limited sample sizes and wide confidence intervals (Appendix I). To test the robustness of our findings, we conducted sensitivity analyses by excluding five studies judged to be at high risk of bias and re-running the NMA. The resulting league tables, forest plots, and SUCRA values were generally similar to those from the main analyses (Appendix II). Excluding high-risk studies did not materially affect the effect estimates or treatment rankings, thereby supporting the robustness of our main conclusions.

## 4 Discussion

This study aimed to rank interventions for improving cardiopulmonary fitness and exercise performance at high altitudes and identify which dietary intervention was most beneficial in modulating the outcome measures of the participants. Our network meta-analysis, based on 20 RCTs involving 329 participants, indicated that different dietary interventions exerted distinct effects on exercise performance at high altitude. Carbohydrate-based strategies showed overall benefits, with carbohydrate plus glutamine supplementation demonstrating advantages in SpO_2_ and RPE, while carbohydrate alone was more consistently supported for VO_2max_ improvement. Iron supplementation ranked highest for HR and HCT, suggesting potential benefits, although these effects did not reach statistical significance. In contrast, interventions such as high-protein, nitrates, antioxidant-rich foods, RC, and d-aspartic acid showed relatively limited effects.

Cardiopulmonary fitness is a core indicator of an individual's exercise performance and adaptability. In high-altitude environments, various physiological factors such as hypoxia significantly affect the autonomic regulation of the heart and lead to a decline in tissue oxygenation, which is reflected by a decrease in SpO_2_ and an increase in HR (33). A change in HR greater than 2.4% is considered the minimally clinically important difference (MCID), while for SpO_2_, a widely accepted MCID is approximately ±4 percentage point (34, 35). VO_2max_ is the gold standard for assessing cardiopulmonary health. Its improvement not only contributes to better cardiopulmonary health but also enhances an individual's exercise performance (36). According to previous research, an increase of 1.0 ml/kg/min in VO_2max_ is associated with a 9% reduction in all-cause mortality risk. Therefore, this threshold was adopted in the present study as the MCID to evaluate the clinical relevance of each intervention's effect (37). RPE is an effective tool for evaluating exercise intensity and physiological load during resistance training. A higher score indicates greater physiological strain caused by exercise and higher cardiopulmonary regulatory pressure (38). For the RPE, a change of 1.5 points is generally considered the MCID, indicating a perceptible and clinically relevant change in exertion levels (34).

Additionally, HCT is positively correlated with SpO_2_ and VO_2max_. This indicator also reflects an individual's oxygen transport and cardiopulmonary adaptation (39). Therefore, the five outcome indicators included in this study provide a comprehensive assessment system for cardiopulmonary fitness, effectively reflecting an individual's physiological status and adaptability during high-altitude exercise.

The network meta-analysis results of this study confirm that CHO supplementation effectively maintains the oxidation rate of energy substrates during high-altitude exercise, significantly increasing VO_2max_ and reducing RPE scores. When combined with glutamine, this intervention ranked highest for improving SpO_2_ and RPE. Our findings are consistent with several previous studies. For example, Caris et al. found that, compared to placebo, CHO supplementation or the combination of CHO and glutamine significantly reduced RPE scores (12). High-altitude training exacerbates the challenges of energy metabolism, and athletes face significantly increased physiological loads compared to sea level, such as shortness of breath, gas exchange disturbances, reduced cardiac output, and central fatigue due to hypoxic responses (40). Therefore, practitioners, coaches, and athletes should not overlook the importance of energy in high-altitude training (41). In lowland areas, CHO supplements have become very common among athletes. According to the FAO Food and Nutrition report, CHO-containing foods are considered to have the most significant impact on exercise performance (42). CHO are the main energy substrate for skeletal muscles during prolonged exercise, and as exercise intensity increases, the proportion of energy supplied by CHO significantly rises (43). During exercise lasting more than 2 h, CHO intake, compared to placebo, prevents hypoglycemia, maintains a higher CHO oxidation rate, and enhances endurance (44). Glutamine, a conditionally essential amino acid, is widely used in sports nutrition. It can influence respiratory function through neuroregulatory mechanisms, helping to restore SpO_2_ (45). The immunoregulatory effects of glutamine maintain immune cell activity, ensuring training continuity, while also delaying exercise-induced fatigue through various mechanisms (46). Furthermore, the hypoxic environment at high altitudes triggers acute immune suppression, and prolonged exercise training further exacerbates this phenomenon, leading to a significant decline in glutamine concentrations in muscles and plasma. This reduction in glutamine leaves immune cells without the energy sources needed for synthesis and nucleotides. As a result, athletes training at high altitudes are at greater risk of infection (47, 48). Studies have shown that the combination of glutamine and CHO improves exercise performance more effectively than glutamine alone (46). The combined supplementation may enhance performance and improve metabolic status after high-intensity training by increasing glycogen reserves, supporting gluconeogenesis, and accelerating recovery (49). Favano et al. (50) supplemented football players undergoing intermittent treadmill training with glutamine peptides and CHO or with CHO alone and observed that compared to CHO supplementation alone, the combination of glutamine and CHO resulted in increased exercise time and distance, while significantly reducing RPE (50). Furthermore, Carvalho-Peixoto et al. (49) found that the combined supplementation of glutamine and CHO effectively reduced blood ammonia accumulation and central fatigue compared to the control group.

The results of the network meta-analysis in this study further confirm that iron supplementation effectively improves tissue oxygen supply by increasing hemoglobin concentration and enhancing blood oxygen-carrying capacity (51). It showed the highest ranking for post-exercise HR and HCT among the interventions assessed. As a core nutrient for oxygen transport and cellular energy metabolism, iron plays a key role in the adaptation process to high-altitude exercise. The hypoxic environment at high altitudes significantly stimulates the secretion of erythropoietin, thereby greatly increasing the body's demand for iron. Athletes, due to exercise-related iron loss pathways (including sweat loss, exercise-induced hemolysis, gastrointestinal bleeding, and menstrual blood loss in female athletes), face a higher risk of iron deficiency (41). Research evidence indicates that daily iron supplementation effectively promotes erythropoiesis during high-altitude adaptation, particularly for athletes with low baseline ferritin levels (52).

Dietary antioxidants are among the most common sports nutrition supplements used to alleviate exercise-induced oxidative stress. Intense exercise and muscle contractions increase the production of reactive oxygen species (ROS) and reactive nitrogen species (RNS), promoting oxidative stress in skeletal muscles. Therefore, endurance athletes commonly take antioxidant supplements to minimize exercise-induced oxidative stress, thereby enhancing recovery and improving athletic performance (53). Additionally, at sea level, dietary nitrates are also widely used as a popular supplement among athletes. Nitrate intake significantly increases the concentration of nitrite (${\text{NO}}_{2}^{-}$) in plasma, which is an important substrate to produce nitric oxide (NO) and a biomarker in its circulation. As a key signaling molecule, NO regulates exercise performance through various physiological mechanisms, including enhancing skeletal muscle contraction efficiency, modulating mitochondrial respiration and energy metabolism, and improving local tissue blood flow. These effects synergistically promote oxygen utilization efficiency and metabolic stability, thereby enhancing exercise endurance and overall performance (54, 55). However, in high-altitude environments, the effects of some antioxidants are not as effective. Our study showed that nitrate supplementation did not demonstrate significant advantages across several indicators. Our previous meta-analysis on the effects of nitrate on cardiopulmonary fitness at high altitudes also showed that while it increased serum nitrite levels, it had no effect on cardiopulmonary fitness (56). Hennis' study similarly indicated that dietary nitrate supplementation did not significantly improve exercise efficiency in high-altitude environments (1). Rhodiola rosea and Cordyceps sinensis are widely found in high-altitude areas in the plateau and mountain regions. Both plants are popular traditional medicines in Europe and Asia. Studies have shown that Rhodiola rosea is often used as an ergogenic aid to enhance endurance performance and antioxidant capacity, while Cordyceps supplementation has been shown to stimulate vasodilation, possibly by stimulating NO release, improving tissue oxygen utilization, and thereby having the potential to enhance endurance performance (17). However, when these two supplements were combined, similar to antioxidant food supplementation, the intervention did not show significant effects. The primary reason for this may be the small sample sizes in the related studies, as most interventions were included in the analysis for only 1–2 indicators, which led to reduced statistical power and made it difficult to fully assess their true effects. This finding is consistent with the studies of Koivisto et al. and Chen et al., which showed no significant effect of antioxidant food supplementation or Rhodiola rosea combined with Cordyceps on HCT (16, 17). This result is similar to high-protein supplementation or D-aspartic acid supplementation, where the effects on high-altitude exercise performance were not significant. Protein is typically considered a low-efficiency fuel source, contributing little to the total energy demands of exercise. For example, Macdermid and Stannard found that cyclists on a high-protein diet took significantly longer to complete a time trial compared to those on a low-protein/high-CHO diet (57). D-aspartic acid may improve muscle function by regulating the hypothalamic-pituitary-gonadal axis and increasing plasma testosterone levels, potentially enhancing exercise performance (58). Płoszczyca et al. found that continuous supplementation of d-aspartic acid did not affect testosterone, cortisol, or hematological responses during training in athletes (14). This may be because the shorter duration of D-aspartic acid supplementation was insufficient to observe changes in hormone secretion.

To the best of our knowledge, this is the first study to systematically assess the ranking of different dietary interventions on high-altitude exercise performance and cardiopulmonary fitness using network meta-analysis methods. The main strength of this study lies in its rigorous methodological design, which comprehensively evaluated multiple key physiological indicators related to high-altitude exercise. The findings clearly identified the three promising nutritional intervention strategies, providing practical guidance for high-altitude trainees. However, there are some limitations. For instance, the overall sample size across the included RCTs was relatively small, with most trials enrolling only a limited number of participants. Such constraints inevitably reduce the statistical power of the analyses and may compromise the stability and precision of the pooled estimates. Under these conditions, both false-negative and false-positive findings become more likely, and some observed effects may reflect random variation rather than consistent clinical benefit. Another key limitation of this review lies in the clinical heterogeneity among the included studies. Specifically, the altitude of exposure varied considerably, ranging from moderate to extreme altitudes, which may have triggered different physiological adaptations. Participants engaged in diverse forms of exercise, such as running, cycling, hiking, and mountaineering, each imposing distinct metabolic and cardiovascular demands. The duration of dietary interventions also varied, from several days to multiple weeks, adding further variability to exposure intensity and adaptation. Additionally, approximately half of the studies were conducted in real high-altitude environments, while the remainder employed simulated hypoxic conditions. These settings differ substantially in terms of environmental stressors such as temperature, terrain, and barometric pressure, which may influence the efficacy of nutritional interventions. Collectively, these variations may have contributed to inconsistencies in effect estimates and complicated the interpretation of outcomes. However, due to the limited number of included studies, formal subgroup analyses could not be performed, representing a constraint in addressing and interpreting potential heterogeneity. Additionally, the current data primarily come from male athletes and active individuals with a training background, which may not fully reflect the impact of gender and training background differences on intervention outcomes. Therefore, the results may be more applicable to trained or active male individuals and may not be fully generalizable to a broader population. Moreover, although SUCRA provides a convenient summary of the relative ranking of different interventions, its interpretation should be approached with caution. SUCRA values are easily influenced by sample size, network structure, and between-study heterogeneity, and they do not directly convey the magnitude or clinical relevance of treatment effects. Some NMAs have attempted to report uncertainty intervals for SUCRA (SUCRA CrIs), but when the analysis is based on only a few small studies, the 95% CrIs are often extremely wide, sometimes spanning the entire range from 0 to 100% (59). This indicates considerable instability and imprecision in the ranking results. Therefore, SUCRA rankings should be regarded as suggestive rather than definitive evidence. Taken together, these limitations mean that the conclusions of the present review should be interpreted with caution, and the overall certainty of evidence is low to moderate. Thus, the effectiveness of targeted dietary interventions in enhancing cardiopulmonary fitness and exercise performance at high altitudes remains a topic of ongoing debate. Future RCTs with larger sample sizes, longer follow-up periods, and more diverse populations (including women and non-athletic individuals), as well as standardized protocols for exercise modality, exposure duration, and supplementation strategies, are urgently needed to confirm and refine these findings, thereby providing more reliable evidence for high-altitude sports nutrition interventions.

## 5 Conclusion

The eight dietary strategies evaluated across 20 randomized controlled trials involving 329 participants, carbohydrate supplementation significantly improved VO_2max_ and reduced RPE, with the observed increase in VO_2max_ exceeding the MCID, indicating potential clinical relevance. Additionally, carbohydrate combined with glutamine ranked first in improving SpO_2_ and RPE, while iron supplementation ranked first for enhancing HR and HCT. However, these latter interventions did not demonstrate statistically significant advantages. Moreover, antioxidant-rich foods, nitrates, high-protein diets, and RC showed limited effects. Overall, carbohydrate-based strategies appear to be the most promising for supporting cardiopulmonary function and exercise performance at high altitude. Although the findings provide a reference for high-altitude sports nutrition strategies, the conclusions are limited by the number of studies and sample heterogeneity. Further high-quality studies are needed to validate these findings and optimize nutritional interventions for high-altitude training populations.

## Data Availability

The original contributions presented in the study are included in the article/Supplementary material, further inquiries can be directed to the corresponding authors.
