# Supplementary material for: Comparative efficacy of different dietary interventions for cardiopulmonary fitness at high altitude: a systematic review and network meta-analysis
Source: Front Nutr. 2025 Nov 4;12:1658950. doi: 10.3389/fnut.2025.1658950 (PMC12624507; doi:10.3389/fnut.2025.1658950)
Supplement: Supplementary file 1 [file Data_Sheet_1.docx]

# Supplementary file. GRADE Quality Assessment for Primary Outcomes.

In this network meta-analysis, we assessed the quality of evidence for five cardiopulmonary outcomes, **maximal oxygen uptake (VO_2max_)**, **rating of perceived exertion (RPE)**, **peripheral oxygen saturation (SpO_2_)**, **heart rate (HR)**, and **hematocrit (HCT),** using the GRADE (Grading of Recommendations, Assessment, Development, and Evaluation) approach. The quality of evidence for **VO_2max_** related comparisons was rated as “high,” indicating overall robust and reliable results despite some risk of bias and inconsistency in individual studies (Table S1). Most comparisons for RPE, SpO_2_, and HR were rated as “moderate” quality, primarily due to the limited number of studies, heterogeneity in intervention types, and some risk of bias or imprecision in individual trials (Tables S2–S4). Notably, the comparison of carbohydrate (CHO) versus high-protein interventions for HR was rated as “very low” quality because of high risk of bias and substantial uncertainty in the effect estimates (Table S4).For the HCT outcome, the small number of included studies and the diversity of interventions led to a sparse network, which prevented the generation of a complete contribution matrix and visualization of GRADE results using the CINeMA (Confidence in Network Meta-Analysis) tool.Overall, the quality of evidence for most outcomes ranged from moderate to high, supporting the robustness of our findings and providing a relatively reliable evidence base for nutritional interventions in high-altitude environments.

Table S1. GRADE Quality Assessment of Evidence for the Effects of VO_2max_

| **Quality assessment** | | | | | | | | **Quality of evidence** |
| --- | --- | --- | --- | --- | --- | --- | --- | --- |
| **Comparison** | **Number of studies** | **Within-study bias** | **Reporting bias** | **Indirectness** | **Imprecision** | **Heterogeneity** | **Incoherence** | **Confidence rating** |
| Antioxidant-rich foods:Placebo | 1 | Some concerns | Low risk | No concerns | Some concerns | Some concerns | Major concerns | Low |
| CHO:Placebo | 2 | Some concerns | Low risk | No concerns | No concerns | No concerns | Major concerns | Moderate |
| Fe:Placebo | 1 | Some concerns | Low risk | No concerns | Major concerns | No concerns | Major concerns | Loew |
| Nitrates:Placebo | 5 | No concerns | Low risk | No concerns | Some concerns | No concerns | Major concerns | Moderate |
| Placebo:RC | 1 | Major concerns | Low risk | No concerns | Major concerns | No concerns | Major concerns | Low |

Note: CHO: Carbohydrate; RC:rhodiola crenulata-and cordyceps sinensis

Table S2. GRADE Quality Assessment of Evidence for the Effects of RPE.

| **Quality assessment** | | | | | | | | **Quality of evidence** |
| --- | --- | --- | --- | --- | --- | --- | --- | --- |
| **Comparison** | **Number of studies** | **Within-study bias** | **Reporting bias** | **Indirectness** | **Imprecision** | **Heterogeneity** | **Incoherence** | **Confidence rating** |
| CHO:CHO+Glutamine | 1 | Some concerns | Low risk | No concerns | Some concerns | No concerns | No concerns | Moderate |
| CHO:Placebo | 5 | Some concerns | Low risk | No concerns | No concerns | Some concerns | No concerns | Moderate |
| CHO+Glutamine:Placebo | 1 | Some concerns | Low risk | No concerns | No concerns | Some concerns | No concerns | Moderate |
| Nitrates:Placebo | 6 | No concerns | Low risk | No concerns | No concerns | Some concerns | No concerns | Moderate |

Note: CHO: Carbohydrate

Table S3. GRADE Quality Assessment of Evidence for the Effects of SpO_2._

| **Quality assessment** | | | | | | | | **Quality of evidence** |
| --- | --- | --- | --- | --- | --- | --- | --- | --- |
| **Comparison** | **Number of studies** | **Within-study bias** | **Reporting bias** | **Indirectness** | **Imprecision** | **Heterogeneity** | **Incoherence** | **Confidence rating** |
| CHO:CHO+Glutamine | 1 | Some concerns | Low risk | No concerns | No concerns | No concerns | No concerns | Moderate |
| CHO:Placebo | 4 | Some concerns | Low risk | No concerns | No concerns | No concerns | No concerns | Moderate |
| CHO+Glutamine:Placebo | 1 | Some concerns | Low risk | No concerns | No concerns | No concerns | No concerns | Moderate |
| Nitrates:Placebo | 8 | Some concerns | Low risk | No concerns | No concerns | No concerns | No concerns | Moderate |

Note: CHO: Carbohydrate.

Table S4. GRADE Quality Assessment of Evidence for the Effects of HR.

| **Quality assessment** | | | | | | | | **Quality of evidence** |
| --- | --- | --- | --- | --- | --- | --- | --- | --- |
| **Comparison** | **Number of studies** | **Within-study bias** | **Reporting bias** | **Indirectness** | **Imprecision** | **Heterogeneity** | **Incoherence** | **Confidence rating** |
| CHO:CHO+Glutamine | 1 | Some concerns | Low risk | No concerns | No concerns | Some concerns | No concerns | Moderate |
| CHO:High Protein | 1 | Major concerns | Low risk | No concerns | Major concerns | No concerns | No concerns | Low |
| CHO:Placebo | 4 | Some concerns | Low risk | No concerns | No concerns | No concerns | No concerns | Moderate |
| CHO+Glutamine:Placebo | 1 | Some concerns | Low risk | No concerns | Some concerns | No concerns | No concerns | Moderate |
| Fe:Placebo | 1 | Some concerns | Low risk | No concerns | Some concerns | No concerns | No concerns | Moderate |
| Nitrates:Placebo | 8 | Some concerns | Low risk | No concerns | Some concerns | No concerns | No concerns | Moderate |

Note: CHO: Carbohydrate; Fe: Iron
