# Supplementary material for: Comparative efficacy of different dietary interventions for cardiopulmonary fitness at high altitude: a systematic review and network meta-analysis
Source: Front Nutr. 2025 Nov 4;12:1658950. doi: 10.3389/fnut.2025.1658950 (PMC12624507; doi:10.3389/fnut.2025.1658950)

# Supplementary file: Sensitivity Analyses for Network Meta-Analyses.

After excluding five studies deemed to be at high risk of bias, a revised network meta-analysis was conducted focusing on five cardiopulmonary outcomes: VO_2max,_ heart rate (HR), peripheral oxygen saturation (SpO_2_), hematocrit (HCT), and rating of perceived exertion (RPE). The intervention networks for each outcome are illustrated in **Figure S1**. Additionally, surface under the cumulative ranking (SUCRA) values were calculated to rank each dietary intervention for each outcome, with results presented in **Figure S2**. Figure S3 and S2 demonstrates that carbohydrate (CHO) supplementation consistently yielded favorable effects, showing significant improvements in VO_2max_ compared with both placebo (standardized mean difference [SMD] = 1.13, 95% credible interval [CrI]: 0.18 to 2.12) and nitrate supplementation (SMD = 1.42, 95% CrI: 0.22 to 2.61). For RPE, CHO alone also outperformed placebo (mean difference [MD] = –0.81, 95% CrI: –1.97 to –0.05). The combination of CHO and glutamine ranked highest for improving RPE and SpO_2_, with SUCRA values of 65.92% and 70.76%, respectively. In addition, iron supplementation ranked highest for improving HR (SUCRA: 61.95%) and HCT (SUCRA: 70.82%).These findings are largely consistent with the results prior to the exclusion of high-risk studies. The exclusion of these studies did not materially alter the effect estimates or the relative ranking of interventions, further supporting the robustness of our primary conclusions.

Figure S1. Network meta-analysis of various interventions on cardiopulmonary fitness.(A) Network of interventions for VO_2max_. (B) Network of interventions for RPE. (C) Network of interventions for SpO_2_. (D) Network of interventions for HR. (E) Network of interventions for HCT. (F) Forest plot displaying weighted standardized mean difference and 95% credible interval for the effect of various interventions versus placebo on VO_2max_ levels. (G) Forest plot displaying weighted mean difference and 95% credible interval for the effect of various interventions versus placebo on RPE levels. (H) Forest plot displaying weighted mean difference and 95% credible interval for the effect of various interventions versus placebo on SpO_2_ levels. (I) Forest plot displaying weighted mean difference and 95% credible interval for the effect of various interventions versus placebo on HR levels. (J) Forest plot displaying weighted mean difference and 95% credible interval for the effect of various interventions versus placebo on HCT levels.


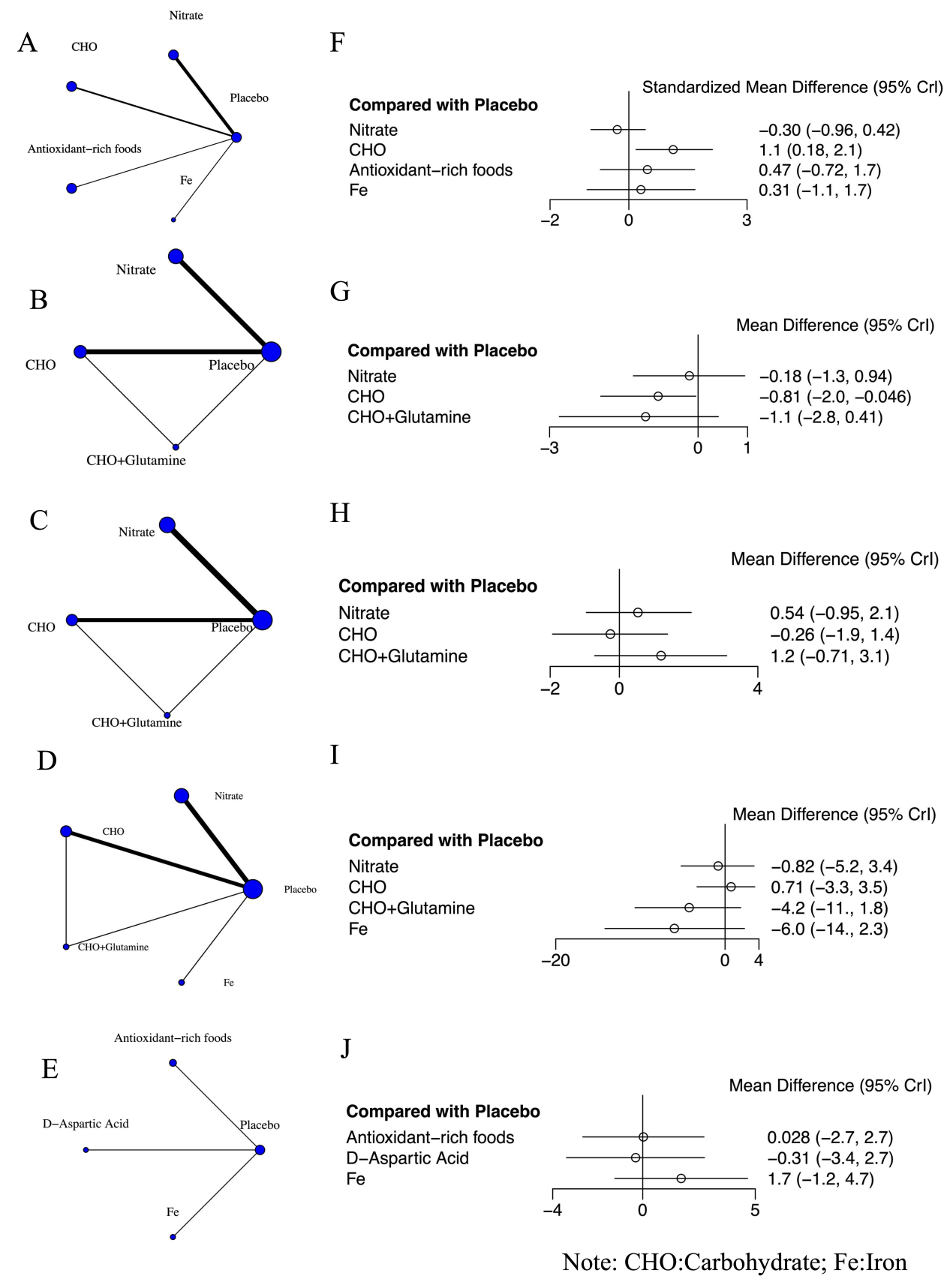


Figure S2. Surface Under the Cumulative Ranking Curve (SUCRA) illustrating the cumulative probability of each intervention being among the best in terms of RPE (A), SpO_2_ (B), HR(C) and HCT(D) levels.


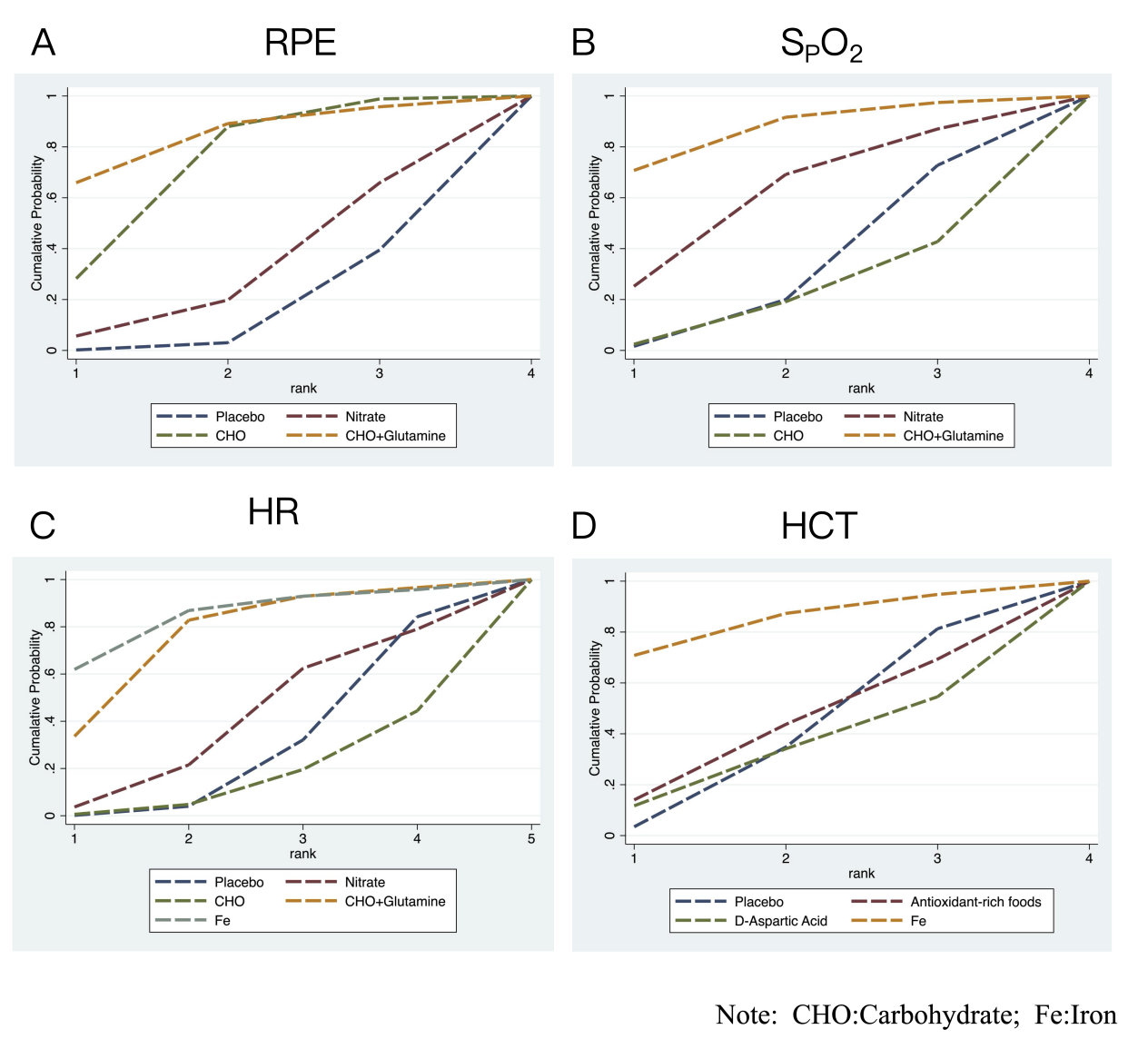


Figure S3. League table displaying pairwise comparisons among various interventions on VO_2max_(A), RPE(B), SpO_2_ (C), HR(D) and HCT(E). Statistically significant differences are bolded (**).


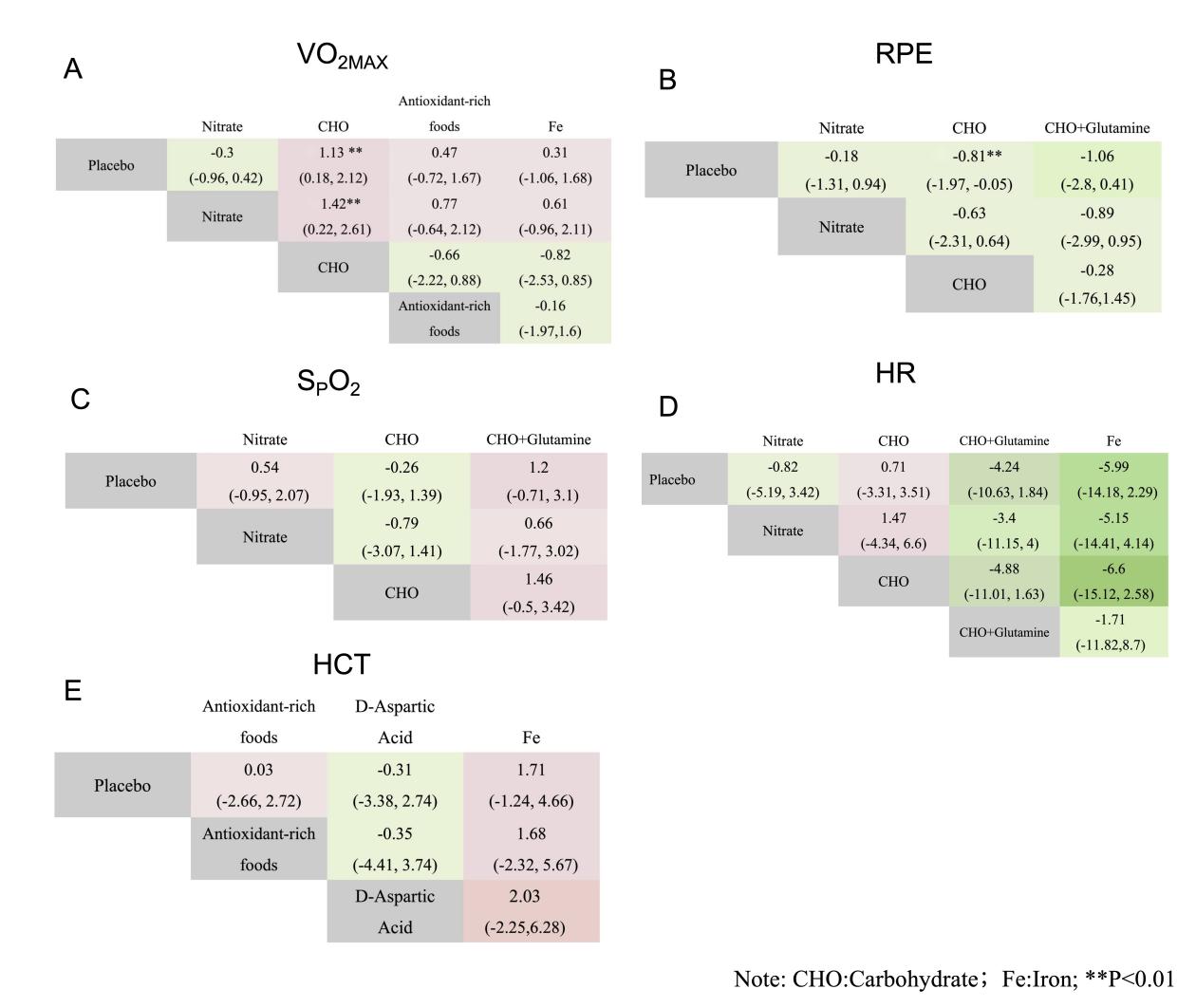

Supplement: Supplementary file 2 [file Data_Sheet_2.docx]
